# Notice to Members P. M. B. A. of Texas

**Published:** 1886-07

**Authors:** F. E. Daniel

**Affiliations:** Secretary and Treasurer


NOTICE TO MEMBERS, P. M. B. A., OF TEXAS.—Our brother, Dr. David Sayres, holder of certificate No. 89, in the PHYSICIANS' MUTUAL BENEFIT ASSOCIATION OF TEXAS, died July 2, 1886, at his home in Bastrop. Assessment No. 2 has been issued to members, calling upon them for $1 each to the fund for the benefit of his family, under the provisions of the Constitution and By-Laws of the Order. Failure to pay within thirty days from the date of notice entails forfeiture of membership and all monies previously paid in, and the erasure from the roll of the name of members so failing to comply with their pledges.

F. E. DANIEL,
Secretary and Treasurer.

